# Secoisolariciresinol Diglucoside Delays the Progression of Aging-Related Diseases and Extends the Lifespan of *Caenorhabditis elegans* via DAF-16 and HSF-1

**DOI:** 10.1155/2020/1293935

**Published:** 2020-07-14

**Authors:** Min Lu, Lin Tan, Xiao-Gang Zhou, Zhong-Lin Yang, Qing Zhu, Jian-Ning Chen, Huai-Rong Luo, Gui-Sheng Wu

**Affiliations:** ^1^Key Laboratory for Aging and Regenerative Medicine, Department of Pharmacology School of Pharmacy, Southwest Medical University, Luzhou, Sichuan 646000, China; ^2^Key Laboratory of Medical Electrophysiology, Ministry of Education, Institute of Cardiovascular Research of Southwest Medical University, Luzhou, Sichuan 646000, China; ^3^Central Nervous System Drug Key Laboratory of Sichuan Province, Luzhou, Sichuan 646000, China

## Abstract

Secoisolariciresinol diglucoside (SDG) is a phytoestrogen and rich in food flaxseed, sunflower seeds, and sesame seeds. Among the beneficial pharmacological activities of SDG on health, many are age related, such as anticancer, antidiabetes, antioxidant, and neuroprotective effects. Thus, we investigated if SDG had an effect on antiaging in *Caenorhabditis elegans* (*C. elegans*). Our results showed that SDG could extend the lifespan of *C. elegans* by up to 22.0%, delay age-related decline of body movement, reduce the lethality of heat and oxidative stress, alleviate dopamine neurodegeneration induced by 6-hydroxydopamine (6-OHDA), and decrease the toxicity of A*β* protein in *C. elegans*. SDG could increase the expression of the downstream genes of DAF-16, DAF-12, NHR-80, and HSF-1 at mRNA level. SDG could not extend the lifespan of mutants from genes *daf-16*, *hsf-1*, *nhr-80*, *daf-12*, *glp-1*, *eat-2*, and *aak-2*. The above results suggested that SDG might enhance the stress resistance, delay the progression of aging-related diseases, and extend the lifespan of *C. elegans* via DAF-16 and HSF-1.

## 1. Introduction

Aging, characterized by a progressive loss of physiological integrity, leads to impaired functioning and increased vulnerability to death [1]. It is highly related to the development of various diseases, such as arteriosclerotic cardiovascular disease, diabetes, Alzheimer's disease, and macular degeneration [2]. Increasingly, researchers are keen to find effective compounds that can promote healthy aging and extend lifespan. Accumulating evidence has demonstrated that functional food and traditional herbs could achieve the benefits, such as royal jelly [3], blueberry [4], *Sophora moorcroftiana* [5], and *Ribes fasciculatum* [6].

**Figure 1:**
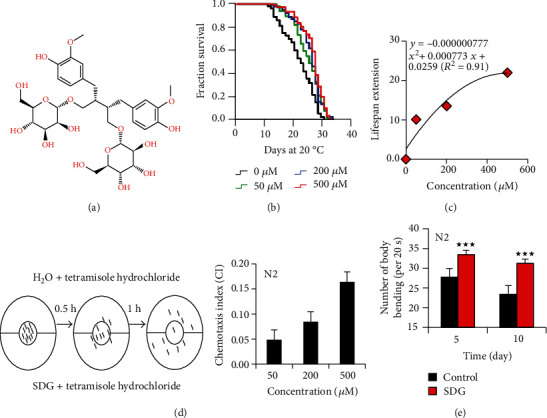
Secoisolariciresinol diglucoside (SDG) extends the lifespan of *C. elegans.* (a) The chemical structure of SDG. (b) The survival of wild-type *C. elegans* (N2) treated with ddH_2_O (control) and 50 *μ*M, 200 *μ*M, and 500 *μ*M of SDG. SDG presented the best effect under the concentration of 500 *μ*M, by extending the lifespan of *C. elegans* up to 22.0%. (c) The lifespan extension of worms under the treatment of SDG with different concentrations. We found the lifespan extension effect of SDG did not show much better at higher dose than 500 *μ*M. (d) The process of chemotaxis assay. Synchronized wild-type worms were placed in the center of assay plates containing SDG plus 2% tetramisole hydrochloride to paralyze the worms on one side of the plate (A side) and H_2_O as solvent control plus 2% tetramisole hydrochloride on the opposite side of the plate (B side). After incubation at 20°C for1 h, CI was scored [CI = (number of worms on A side − number of worms on B side)/number of worms on A side + number of worms on B side], where CI > 0. It means that *C. elegans* do not use chemotaxis to avoid SDG, suggesting SDG has no lethal stimulus to worms. (e) Body bending of wild-type *C. elegans* (N2) treated with or without 500 *μ*M of SDG. Worms treated with 500 *μ*M of SDG showed significantly delayed age-dependent decline of body bending. The columns showed the mean value of one independent experiment with error bars representing SEM. A statistically significant *p* value was calculated by the *t*-test or log-rank test; ^★★★^*p* < 0.0001. The results of three repeated experiments and their statistical analysis are summarized in supplementary Tables S1-S4.

Flaxseed, used as both daily food and medicine, is rich in omega 3 fatty acid, alpha-linolenic acid, secoisolariciresinol diglucoside (SDG), and fiber. Flaxseed protects the health of organisms via the bioactivity of its contents, such as anti-inflammatory action, antioxidative capacity, and lipid-modulating properties [7]. SDG (Figure 1(a)) is a phytoestrogen highly rich in flaxseed. Studies under various cellular and animal models have demonstrated that SDG has potent bioactivity in reducing the risk of cancer, ameliorating oxidative stress, preventing diabetes, and protecting from neurodegenerative disease, such as Alzheimer's disease and Parkinson's disease [8, 9]. SDG could reduce tumor growth via inhibiting the activity of nuclear factor kappa-B in murine E0771 cells (a model of triple-negative breast cancer) [10]. SDG is also a potent angiotensin-converting enzyme (ACE) inhibitor [11]. Additionally, SDG could induce the expression of adiponectin and affect lipid metabolism in diet-induced fat mice [12]. Given that bioactivities of SDG are associated with aging, we are wondering if SDG could extend the lifespan of *Caenorhabditis elegans* (*C. elegans*). Because of its short lifespan, amenability to genetic manipulation, clear genetic background, and a proportion of homologous genes in humans, *C. elegans* is an excellent model for screening chemicals with the longevity modulation effect [13]. Thus, we found that SDG could extend lifespan, improve stress resistance, and improve the symptoms of geriatric diseases in *C. elegans*.

## 2. Materials and Methods

### 2.1. Chemicals and Strains

All strains were obtained from *Caenorhabditis* Genetics Center (CGC) and maintained at an appropriate temperature as described previously [14] unless otherwise stated. Strains used in this study were as follows: N2 (Bristol, wild-type), DA1116 *eat-2 (ad1116)*, MQ887 *isp-1 (qm150)*, CF1038 *daf-16 (mu86)*, RB754 *aak-2 (ok524)*, PS3551 *hsf-1 (sy441)*, AA89 *daf-12 (rh274)*, CF1903 *glp-1 (e2144)*, BX165 *nhr-80 (tm1011)*, BZ555 *egIs1 (dat-1::gfp)*, CF1553 *muIs84 (sod-3::gfp)*, SJ4100 *zcIs13V (hsp-6::gfp)*, SJ4005 *zcIs4V (hsp-4::gfp)*, and CL4176 *[smg-1 (cc546ts); dvIs27(pAF29+pRF4)]*. All strains were maintained and grown on NGM plates seeded with *E. coli* OP50. The CL4176 strain, the temperature-sensitive mutant strain, was maintained at 15°C. For CF1903 strain, L1 larvae were cultured at 20°C for 6 hours, then transferred to 25°C until they develop into late L4 larvae or early adults [15] and subsequently transferred to the experimental plate and cultured at 20°C for lifespan assay.

Secoisolariciresinol diglucoside (SDG) was purchased from Shanghai Yuanye Bio-Technology Co. Ltd. and dissolved in ddH_2_O. NGM plates containing SDG were equilibrated overnight before use.

### 2.2. Lifespan Assay

All strains were cultured for 2-3 generations without starvation. All lifespan assays were conducted at 20°C, unless otherwise stated. At least 60 synchronized L4 larvae or young adult worms were transferred to NGM plates (9 cm, diameter) containing inactivated OP50 (60°C for 35 min). 50 *μ*M of 5-fluoro-2′-deoxyuridine (FUDR, Sigma) was used to prevent self-fertilization [16]. The day L4 larvae or young adult worms were transferred to the experimental plate is defined as experiment day 0. To ensure that SDG retained its potency throughout the entire experiment, worms were transferred to fresh plates with or without SDG every other day. Worms that did not respond to a mechanical stimulus were scored as dead. Meanwhile, worms that crawled off the plate, displayed extruded internal organs, or died because of hatching progeny inside the uterus were censored [17]. Statistical analyses were carried out using SPSS packages. Kaplan-Meier lifespan analysis was carried out, and a statistically significant *p* value was calculated using a log-rank test.

### 2.3. Chemotaxis Assays

Many organisms use chemotaxis to seek out food sources, avoid noxious substances, and find mates. *C. elegans* has impressive chemotaxis behavior. To analyze whether *C. elegans* use chemotaxis to avoid SDG, we performed chemotaxis assays (see Figure 1(d)) using synchronized late L4 larvae or young adult wild-type N2 worms as described previously [18, 19]. About 200 worms were placed in the center of an assay NGM plate (diameter: 3.5 cm), which contain 50, 200, or 500 *μ*M of SDG plus 2% tetramisole hydrochloride to paralyze the worms on one side of the plate (A side) and H_2_O as solvent control plus 2% tetramisole hydrochloride on the opposite side of the plate (B side). After incubation at 20°C for 1 h, chemotaxis index (CI) was scored [CI = (number of worms on A side − number of worms on B side)/number of worms on A side + number of worms on B side].

### 2.4. Phenotypic Assays

In body bending assay, synchronized late L4 larvae or young adult worms (wild-type) were transferred to each plate with or without 500 *μ*M of SDG and cultured for 5 or 10 days at 20°C. Before the body bending assay, worms were gently transferred to the plate with a drop of M9 buffer and left to acclimatize for 15 seconds at room temperature, and then observed under a stereomicroscope for 20 seconds to score the bending activity of the body. The assay was repeated at least three times. A statistically significant *p* value was determined by a *t*-test.

In thermos-tolerance assay, synchronized late L4 larvae or young adult worms (wild-type) were transferred to fresh NGM plates with or without 500 *μ*M of SDG and cultured for 10 days at 20°C. Then, the temperature was upshifted to 35°C, and dead worms were counted every 2 hours. Worms were considered dead when they failed to respond to a touch with a platinum wire. The assay was repeated at least three times. A statistically significant *p* value was determined by the log-rank test.

In oxidative stress assay, synchronized late L4 larvae or young adult worms (wild-type) were transferred to the plates with or without 500 *μ*M of SDG and cultured for 10 days at 20°C. Then, worms were transferred to NGM plates containing 20 mM of paraquat (Sigma), and dead worms were counted every 12 hours. Worms were considered dead when they failed to respond to a touch with a platinum wire. The assay was repeated at least three times. A statistically significant *p* value was determined by the log-rank test.

In pharyngeal pumping assay, synchronized late L4 larvae or young adult worms (wild-type) were transferred to fresh NGM plates with or without 500 *μ*M of SDG and cultured for 5 or 10 days at 20°C. Then, worms were transferred to fresh NGM plates and were observed under a stereomicroscope for 20 seconds to score the pumping activity of the pharynx. The assay was repeated at least three times. A statistically significant *p* value was determined by the *t*-test.

### 2.5. Fluorescence Intensity Quantification Assay

In fluorescence intensity quantification assay, we used mutant strains SJ4005, SJ4100, and CF1553 to analyze whether SDG could affect the expression of HSP-4, HSP-6, and SOD-3, respectively. Each plate contains 50 *μ*M of FUDR to inhibit egg hatching. About 100 late L4 larvae or young adults (mutants) were transferred to plates with or without 500 *μ*M of SDG and cultured at 20°C with inactivated OP50 until the 10th day. Then, we took pictures using a fluorescence microscope (Leica DM6B). Lastly, we used ImageJ to quantify the intensity of fluorescence. Live images were taken from at least 30 worms per group. Three independent replicate experiments were performed. A statistically significant *p* value was calculated by the *t*-test.

### 2.6. Measurement of Reactive Oxygen Species (ROS)

In this assay, synchronized late L4 larvae or young adult worms (wild-type) were transferred to the plates with or without 500 *μ*M of SDG and cultured for 5 or 10 days at 20°C. Then, worms were collected and washed with M9 to a 1.5 ml EP tube 2-3 times and stained with 2′-7′-dichlorofluorescein diacetate (H2DCF-DA) (1 : 1000) and incubated in the dark for 35 min, as described in the staining kit [20]. Pictures were taken by a fluorescence microscope (Leica DM6B). Live images were taken from at least 30 worms per group. ImageJ was used to analyze the gray value. The staining assay contained at least three independent repeat experiments. A statistically significant *p* value was calculated by the *t*-test.

### 2.7. Paralysis Assay

Transgenic strain CL4176 carrying human amyloid-*β* protein was used in the paralysis assay [21]. Worms were maintained at 15°C until L3 larvae stage. Then, worms were transferred to fresh NGM plates with or without 500 *μ*M of SDG and incubated at 25°C to induce the expression of A*β*1-42. We used a platinum wire pick to tap twice on the head of worms, and worms that swung their heads but not the rest of their body were considered paralyzed. Statistical analyses were carried out using SPSS packages. Kaplan-Meier lifespan analysis was carried out, and a statistically significant *p* value was calculated using the log-rank test.

### 2.8. Progeny Viability Assay

At least 30 synchronized late L4 larvae or young adult worms (wild-type) were transferred to fresh NGM plates with or without 500 *μ*M of SDG individually and allowed to lay eggs for 24 h. Then, worms were transferred to a fresh NGM plate again and the remaining eggs were hatched and grown for another 48 h at 20°C. The progeny generated in the remaining plate was counted. Adult worms were transferred to a fresh NGM plate every day until no progeny was produced from each worm. The assay included at least three independent replicate experiments. A statistically significant *p* value was calculated by the *t*-test.

### 2.9. Oil Red O Staining Assay

We use Oil Red O Staining to quantify lipid abundance and evaluate lipid distribution in *C. elegans* [22]. About 100 late L4 larvae or young adult worms (wild type) were transferred to fresh NGM plates with or without 500 *μ*M of SDG and cultured at 20°C for 10 days (worms were transferred to new plates every other day). Then, worms were collected and washed with M9 to a 1.5 ml EP tube 2-3 times, and stained with the oil red O staining kit. Pictures were taken by a fluorescence microscope (Leica DM6B). Live images were taken from at least 30 worms per group. ImageJ was used to analyze the gray value. The Oil Red O Staining experiment contained at least three independent repeat experiments. A statistically significant *p* value was calculated by the *t*-test.

### 2.10. Dopaminergic Neurodegeneration Assay

We used the transgenic strain BZ555 in the dopaminergic neurodegeneration assay. As previously described [23], synchronized late L3 larva worms (the BZ555 strain) were transferred to buffer containing 50 mM of 6-OHDA and 10 mM of ascorbic acid, incubated for one hour at 20°C, and mixed gently every 10 min. After one hour, worms were washed three times with M9 buffer and then incubated with or without SDG for 72 hours at 20°C. Lastly, we took pictures to analyze the dopaminergic neurodegeneration by a fluorescence microscope (Leica DM6B). Live images were taken from at least 30 worms per group. Worms could be scored as neurodegeneration when any part of the dendrite was absent or the cell body shrank [24]. We used ImageJ to quantify the intensity of fluorescence located at the dendrite of worms. At least three independent replicate experiments were performed. A statistically significant *p* value was calculated by the *t*-test.

### 2.11. Quantitative RT-PCR Assay

About 2,000 synchronized young adult worms were transferred to six NGM plates (9 cm, diameter) with or without 500 *μ*M of SDG and maintained at 20°C for 24 hours. Total RNA was extracted using RNAiso Plus (Takara) and converted to cDNA using a High Capacity cDNA Reverse Transcription Kit (Applied Biosystems). The quantitative RT-PCR was performed using the Power SYBR Green PCR Master Mix (Applied Biosystems) in a QuantStudio 6 Flex system. The relative expression levels of genes were carried out using the 2^−*ΔΔ*CT^ method and normalized to the expression of gene *cdc-42* [17]. Statistically significant *p* values were calculated using the *t*-test. Partial quantitative RT-PCR primers were as follows:

1. *cdc-42*: 5′-CTGCTGGACAGGAAGATTACG-3′ (F) and 5′-CTCGGACATTCTCGAATGAAG-3' (R)
2. *hsp-60*: 5′-AAGGATATGGGAATTGCGACGGGA-3′ (F) and 5′-TGTGCTCGATTCGCTTCTCGATCT-3′ (R)
3. *hsp-16.2*: 5′-CTGCAGAATCTCTCCATCTGAGTC-3′ (F) and 5′-AGATTCGAAGCAACTGCACC-3′ (R)
4. *sod-3*: 5′-AGCATCATGCCACCTACGTGA-3′ (F) and 5′-CACCACCATTGAATTTCAGCG-3′ (R)
5. *fard-1*: 5′-GGGTTTTTGGGAAAGGTGAT-3′ (F) and 5′-CCACCGATTGCTTTCAATTT-3′ (R)

### 2.12. Statistical Analysis

Data are presented as the means ± SEM unless specifically indicated. Statistical analyses included the *t*-test or log-rank test. All figures were generated using GraphPad Prism 6, SPSS, or MS Office.

## 3. Results

### 3.1. Secoisolariciresinol Diglucoside (SDG) Extends the Lifespan of C. elegans

To investigate the effect of SDG on lifespan, we treated worms (wild-type) with different concentrations of SDG ranging from 0 to 500 *μ*M. Our results showed that SDG extended the lifespan of *C. elegans* in a dose-dependent manner and presented the best effect under the concentration of 500 *μ*M, by extending the lifespan of *C. elegans* up to 22.0% (Figure 1(b)). To analyze the lifespan extension effect of SDG at higher dose, we did the curve fitting to extrapolate a top of the dose response. We found the lifespan extension effect of SDG did not show much better at higher dose than 500 *μ*M (Figure 1(c)). *C. elegans* has impressive chemotaxis behavior to avoid noxious substances. To analyze whether SDG has a harmful stimulation or even a fatal effect on worms at high dose, we have performed chemotaxis assays. We found worm did not avoid SDG (Figures 1(d) and S1). The body movement of *C. elegans* decreases with aging [25]. To investigate whether SDG could delay the age-related decline of phenotypes, the body movement of worms was monitored and analyzed. In this study, we selected the frequency of body bending as a measurement of nematode movement behavior as the previous study [26]. Our results showed that body bending declined progressively with or without SDG during aging in *C. elegans*. However, upon treatment with SDG, body bending was significantly increased at 5 days and 10 days of adults (*p* < 0.0001, Figure 1(e)).

### 3.2. SDG Improves the Stress Resistance of C. elegans

**Figure 2:**
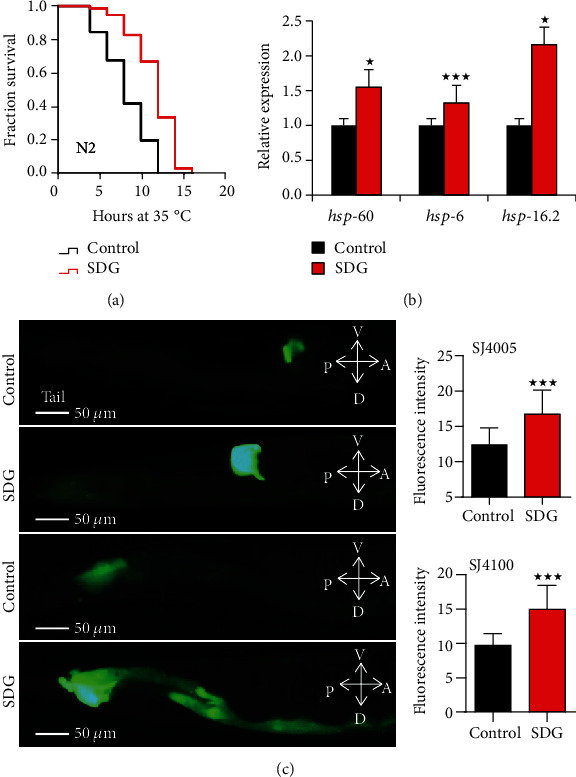
SDG improves the heat stress resistance of *C. elegans.* (a) The survival of wild-type *C. elegans* (N2) treated with or without 500 *μ*M of SDG at 35°C. SDG can significantly extend the lifespan by up to 31.6% under heat stress (*p* < 0.0001, log-rank test). (b) The mRNA level of heat-shock related genes in wild-type *C. elegans* (N2) treated with or without 500 *μ*M of SDG. The expression of the heat-shock related genes showed a significant difference (*p* < 0.05, two-tailed *t*-test). (c) The pictures of green fluorescent at tail in *C. elegans* SJ4005 *zcIs4V (hsp-4::gfp)* and SJ4100 *zcIs13V (hsp-6::gfp)* treated with or without 500 *μ*M of SDG. Worms were observed and photographed using a fluorescent microscope (Leica DM6B) with appropriate filter sets for GFP (magnification: 200x). The quantification of fluorescence intensity showed that the expression of HSP-4::GFP and HSP-6::GFP was significantly increased with SDG treatment (*p* < 0.0001, two-tailed *t*-test). A statistically significant *p* value was calculated by the *t*-test or log-rank test; ^★^*p* < 0.05; ^★★★^*p* < 0.0001. The results from repeated experiments and the statistical details are summarized in supplementary Tables S5–S7.

**Figure 3:**
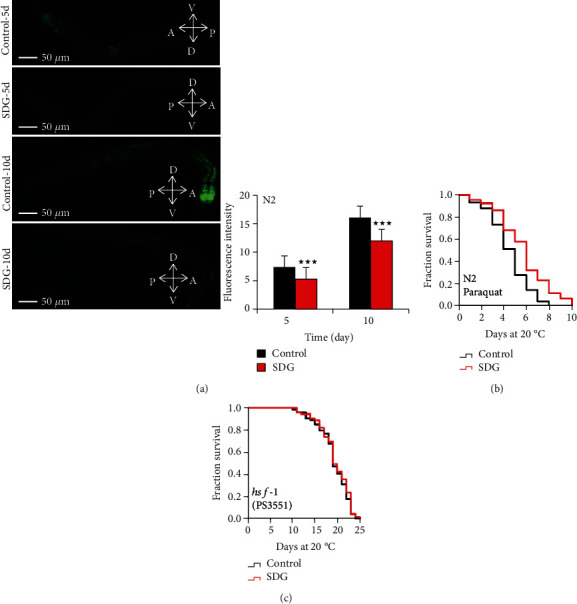
SDG improves the oxidative stress resistance of *C. elegans.* (a) The measurement of reactive oxygen species (ROS). Worms were observed and photographed using a fluorescent microscope (Leica DM6B) with appropriate filter sets for GFP (magnification: 100x). The quantification of fluorescence intensity showed that SDG could decrease the level of ROS in worms (*p* < 0.0001, two-tailed *t-*test). (b) The survival curve of wild-type *C. elegans* (N2) treated with or without 500 *μ*M of SDG under oxidative stress induced by 20 mM of paraquat. SDG can significantly extend the lifespan of *C. elegans* by up to 27.0% (*p* < 0.0001, log-rank test). (c) The survival of *hsf-1* mutant strain PS3551 treated with or without 500 *μ*M of SDG at 20°C. SDG could not further extend the lifespan of *hsf-1* mutant. A statistically significant *p* value was calculated by the *t*-test or log-rank test; ^★★★^*p* < 0.0001. The results from repeated experiments and the statistical details are summarized in supplementary Tables S5 and S8.

Since an increased lifespan is usually closely associated with enhanced survival under stress conditions [27, 28], we further analyzed if SDG could improve the resistance of *C. elegans* to heat stress. In heat stress assay, N2 worms were treated with 500 *μ*M of SDG for 10 days at 20°C and then were transferred to 35°C. SDG treatment extended the lifespan of *C. elegans* by up to 31.6% at 35°C (*p* < 0.0001, Figure 2(a)). Heat shock proteins encoding genes *hsp-16.2*, *hsp-4*, *hsp-6*, and *hsp-60* are associated with heat stress resistance. To test if SDG could improve heat stress resistance of *C. elegans*, we analyzed the mRNA level of heat stress-related genes in *C. elegans*. SDG increased the mRNA level of genes *hsp-16.2*, *hsp-60*, and *hsp-6* (*p* < 0.05, Figure 2(b)). Furthermore, SDG increased the expression of HSP-6::GFP and HSP-4::GFP fusion protein in transgenic strains SJ4100 and SJ4005, respectively (*p* < 0.0001, Figure 2(c)). Meanwhile, we also performed the assay to measure the level of ROS in *C. elegans*. Our results showed that SDG decreased the level of ROS in worms (*p* < 0.0001, Figure 3(a)). Further, to analyze if SDG-treated worms were resistant to oxidative stress, worms were exposed to paraquat, an intracellular free radical-generating compound inducing acute oxidative stress. SDG improved the survival rate of worms and extended their lifespan by up to 27.0% (*p* < 0.0001, Figure 3(b)).

HSF-1, an essential nuclear protein, is associated with heat-shock response and aging in *C. elegans* [29]. To test if SDG could prolong the lifespan of *C. elegans* via HSF-1, we analyzed the lifespan of *hsf-1* mutant strain PS3551 treated with or without SDG. SDG could not significantly extend the lifespan of *hsf-1* mutant (*p* > 0.05, Figure 3(c)). So our results showed that SDG could increase stress resistance to heat and oxidative stress and may extend lifespan via HSF-1 in *C. elegans.*

### 3.3. SDG Protects DA Neurons from 6-OHDA-Induced Neurodegeneration and Decreases A*β*-Induced Toxicity in C. elegans

**Figure 4:**
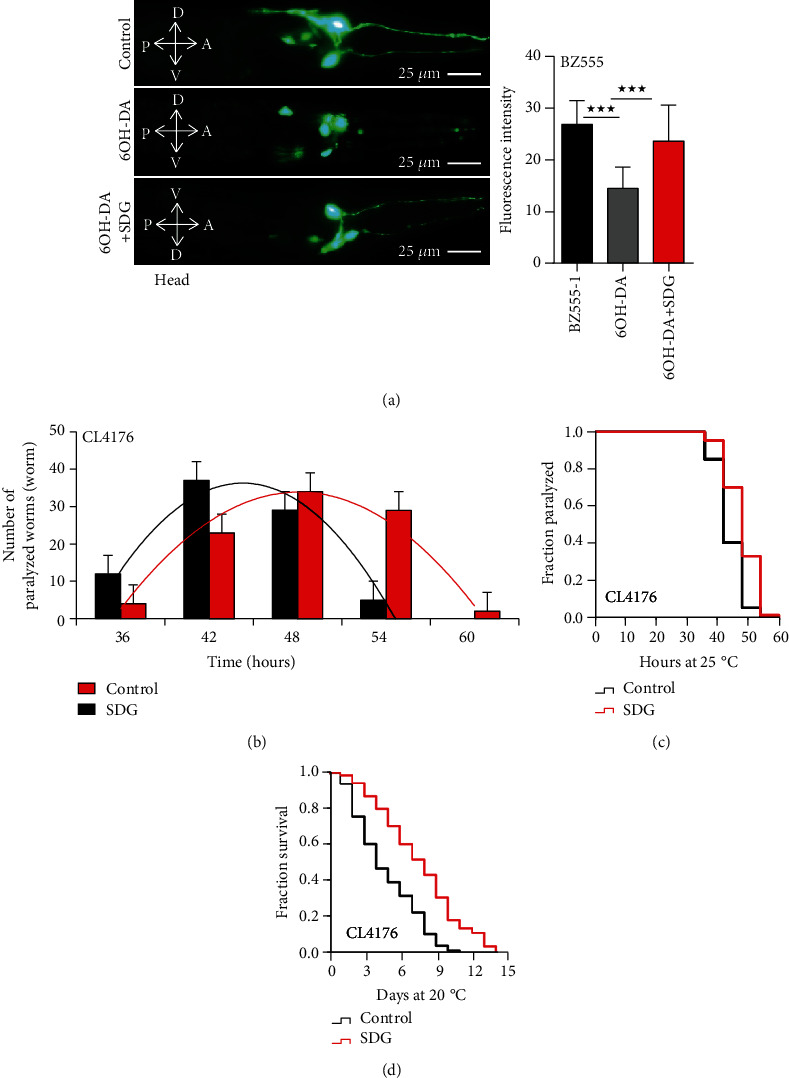
SDG protects DA neurons from 6-OHDA-induced neurodegeneration and decreases A*β*-induced toxicity in *C. elegans*. (a) The result of dopaminergic neurodegeneration assay. Worms were observed and photographed using a fluorescent microscope (Leica DM6B) with appropriate filter sets for GFP (magnification: 400x). SDG improved the shrinkage of neuronal cell bodies caused by 6-OHDA. The fluorescent intensity of DAT-1::GFP at neuronal dendrite was decreased due to 6-OHDA in BZ555 mutant, which was improved with SDG treatment significantly; *p* < 0.0001. (b, c) The results of paralysis assays using CL4176 strain treated with or without 500 *μ*M of SDG. The number of worms paralyzed every six hours was fitted by normal distribution curves to indicate the peak period of paralysis, while Kaplan-Meier survival curves showed the onset of paralysis of CL4176 strain. SDG significantly delayed the onset of paralysis of the CL4176 strain caused by A*β*-induced toxicity at 25°C (*p* < 0.0001, log-rank test). (d) The mean lifespan of CL4176 strain treated with or without 500 *μ*M of SDG at 25°C. SDG extended the lifespan of CL4176 by up to 57.0% (*p* < 0.0001, log-rank test). A statistically significant *p* value was calculated by the *t*-test or log-rank test; ^★★★^*p* < 0.0001. The results from three repeated experiments and the statistical details are summarized in supplementary Tables S5, S7, and S9.

Parkinson's disease (PD) and Alzheimer's disease (AD) are neurodegenerative disorders in the elderly population. Dopaminergic (DA) neuron loss and *α*-synuclein aggregation may lead to PD [30]. By administration of 6-hydroxydopamine (6-OHDA), a neurotoxic agent, the degeneration of GFP-tagged DA neurons could be observed in *C. elegans* transgenic mutant strain BZ555 [30]. To identify whether SDG could protect DA neurons from 6-OHDA-induced neurodegeneration, we quantified the fluorescence intensity of neuronal dendrites in the strain BZ555. We found that 6-OHDA-treated worms expressed lower intensity of GFP compared with non-6-OHDA-treated worms. 6-OHDA-treated worms, after being incubated with SDG, showed recovered intensity of GFP intensity. Meanwhile, we found that SDG improved the shrinkage of neuronal cell bodies caused by 6-OHDA. It suggested that SDG could protect DA neurons from 6-OHDA-induced neurodegeneration in *C. elegans* (Figure 4(a)).

The cholinergic dysfunction and oxidative stress caused by A*β*-induced neurotoxicity are key pathological events in the development of AD [31]. The transgenic mutant strain CL4176, *C. elegans* models of AD, expresses human muscle-specific A*β*1-42 in the cytoplasm of body wall muscle cells and paralyzes at 25°C [31]. To identify whether SDG could affect the A*β*1-42-induced toxicity in *C. elegans*, we performed the paralysis and lifespan assay with the strain CL4176. Our results showed that SDG decreased the A*β*-induced paralysis in the transgenic strain CL4176 (*p* < 0.0001, Figures 4(b) and 4(c)) and extended the lifespan of the strain CL4176 by up to 57.0% at 25°C (Figure 4(d)), suggesting that SDG decreased the A*β*1-42-induced toxicity in *C. elegans*.

### 3.4. SDG Extends the Lifespan of C. elegans via DAF-16

**Figure 5:**
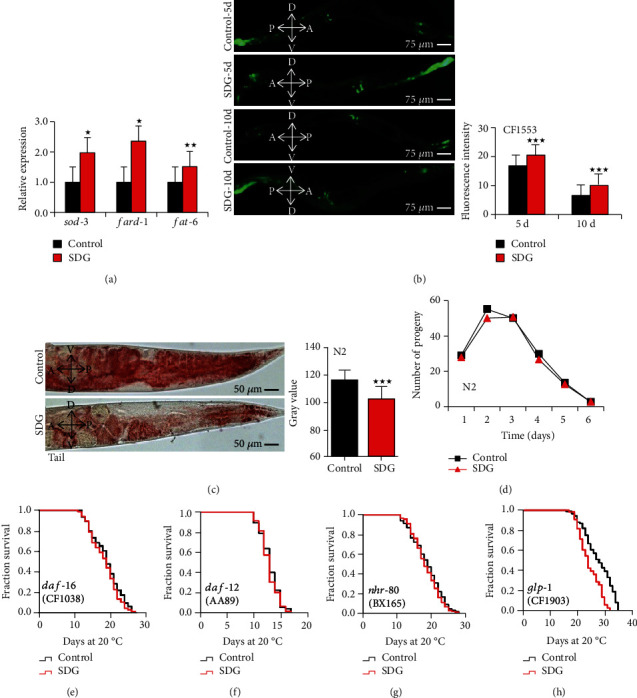
SDG extends the lifespan of *C. elegans* via DAF-16. (a) The mRNA level of genes downstream of DAF-16, DAF-12, and NHR-80 in wild-type *C. elegans* (N2) treated with or without 500 *μ*M of SDG. SDG significantly increased the expression of genes *sod-3*, *fard-1*, and *fat-6* at mRNA level in worms (*p* < 0.05, two-tailed *t*-test). (b) The quantification of fluorescence intensity of SOD-3::GFP in CF1553 *muIs84 (sod-3::gfp)* treated with or without 500 *μ*M of SDG. Worms were observed and photographed using a fluorescent microscope (Leica DM6B) with appropriate filter sets for GFP (magnification: 100x). SDG significantly increased the expression of protein SOD-3 in *C. elegans* (*p* < 0.0001, two-tailed *t-*test). (c) The fat content at tail of wild-type *C. elegans* (N2) treated with or without 500 *μ*M of SDG. Worms were observed and photographed using a fluorescent microscope (Leica DM6B) (magnification: 200x). SDG significantly reduced the fat content in *C. elegans*. (d) The progeny production of per wild-type *C. elegans* (N2) treated with or without 500 *μ*M of SDG. SDG did not affect the reproductive ability of worms. (e) The mean lifespan of *daf-16* mutant strain CF1038 treated with or without 500 *μ*M of SDG at 20°C. (f) The mean lifespan of *daf-12* mutant strain AA89 treated with or without 500 *μ*M of SDG at 20°C. (g) The mean lifespan of *nhr-80* mutant strain BX165 treated with or without 500 *μ*M of SDG at 20°C. (h) The mean lifespan of *glp-1* mutant strain CF1903 treated with or without 500 *μ*M of SDG at 20°C. SDG could not extend the lifespan of *daf-16*, *daf-12*, *nhr-80*, and *glp-1* mutant strains (*p* > 0.05, log-rank test). A statistically significant *p* value was calculated by the *t*-test or log-rank test; ^★^*p* < 0.05; ^★★^*p* < 0.001; ^★★★^*p* < 0.0001. The results from repeated experiments and the statistical details are summarized in supplementary Tables S5, S6, and S10-S12.

Signals from reproductive tissues regulate the lifespan of many organisms [32]. Germline signals regulate stress resistance, lipid metabolism, and lifespan through nuclear transcription factors DAF-16, DAF-12, and NHR-80 [33–35]. Here, to test if SDG could affect the transcriptional activity of these transcription factors, we analyzed the mRNA level of genes downstream of DAF-16, DAF-12, and NHR-80 in *C. elegans*, including *sod-3*, *fard-1*, and *fat-6* [36], respectively. As shown in Figure 5(a), SDG increased the mRNA level of *sod-3*, *fard-1*, and *fat-6* (*p* < 0.05). The expression of SOD-3::GFP fusion protein was significantly increased in *C. elegans* transgenic strain CF1553 treated with SDG (*p* < 0.0001, Figure 5(b)). NHR-80-targeted gene *fat-6* regulates the lipid metabolism of *C. elegans* [37]. To identify whether SDG could affect lipid metabolism, we analyzed the total fat content by Oil Red O Staining. SDG significantly reduced the total fat content (*p* < 0.0001, Figure 5(c)). Lipid metabolism was reported to be associated with reproductive ability and age in *C. elegans* [38]. To analyze if SDG could affect the reproductive ability of *C. elegans*, we scored the number of progeny by the spawning assay. However, SDG did not significantly decrease the number of progeny in *C. elegans* (*p* > 0.05, Figure 5(d)).

Further, our results showed that SDG could not extend the lifespan of *daf-16* mutant strain CF1038, *daf-12* mutant strain AA89, and *nhr-80* mutant strain BX165 (Figures 5(e)–5(g)). The *glp-1* mutant strain CF1903 shows prolonged lifespan due to failed germline proliferation at a specific temperature, which is dependent on the transcriptional activities of DAF-16, DAF-12, and NHR-49 [39]. Thus, to analyze whether SDG could extend the lifespan of worms depending *glp-1*, we measured the lifespan of the null mutants of *glp-1* with SDG treatment. SDG could not extend the lifespan of *glp-1* mutant strain CF1903. (Figure 5(h)).

### 3.5. SDG Could Not Extend the Long-Lived C. elegans Mutants from Genes Involved in Energy Processing

**Figure 6:**
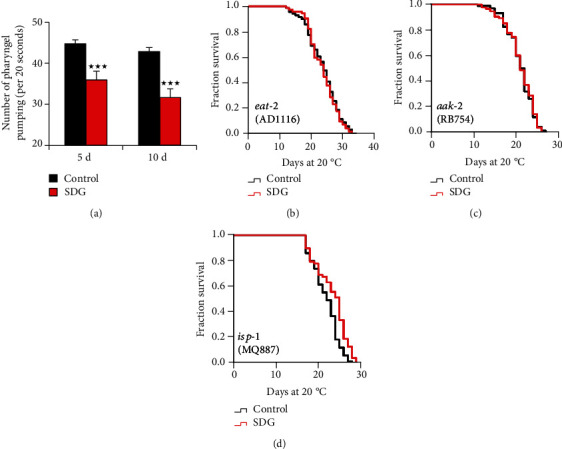
SDG could not extend the long-lived *C. elegans* mutants from genes involved in energy processing. (a) The pharyngeal pumping of wild-type *C. elegans* (N2) treated with or without 500 *μ*M of SDG. The pharyngeal pumping of *C. elegans* was significantly reduced by SDG (*p* < 0.0001, two-tailed *t-*test). (b) The mean lifespan of *eat-2* mutant strain DA1116 treated with or without 500 *μ*M of SDG at 20°C. (c) The mean lifespan of *aak-2* mutant strain RB754 treated with or without 500 *μ*M of SDG at 20°C. (d) The mean lifespan of *isp-1* mutant strain MQ887 treated with or without 500 *μ*M of SDG at 20°C. SDG could not further extend the mean lifespan of these mutants expect for the *isp-1* mutant strain MQ887. A statistically significant *p* value was calculated by the *t*-test or log-rank test; ^★★★^*p* < 0.0001. The results from three repeated experiments and the statistical details are summarized in supplementary Tables S5 and S13.

Dietary restriction (DR) could extend the lifespan in many organisms, from *C. elegans* to monkeys [40, 41]. Reducing pharyngeal pumping often leads to decreased food intake in *C. elegans* [42]. Thus, to test if SDG could affect the food intake of *C. elegans*, the pharyngeal pumping of worms was monitored. We found that SDG-treated worms exhibited significantly slower pharyngeal pumping than nontreated worms, suggesting that SDG affected the food intake of worms (Figure 6(a)). When the food intake of organisms from yeast to mammals is reduced (DR), they live longer than organisms fed a normal diet [43]. Feeding-defective *eat-2* mutant strain is often used as a long-lived DR model [44]. To test if SDG could extend the lifespan of worms in DR pathways, we measured the lifespan of *eat-2* mutant with or without SDG treatment. SDG could not extend the lifespan of long-lived *eat-2* mutant strain DA1116 (Figure 6(b)). The low-energy sensing AMP-activated protein kinase AMPK/*aak-2* could be activated by a low energy level in a DR regimen [45]. Gene *isp-1* encodes Rieske iron-sulfur polypeptide 1, which is involved in mitochondrial electron transport chain and can activate the AMPK pathway by decreasing the level of ATP [46]. Thus, we could think that *aak-2* and *isp-1* were associated with signals in the DR pathway. To analyze whether SDG could extend the lifespan of worms depending *aak-2* and *isp-1*, we measured the lifespan of *aak-2* and *isp-1* mutant with or without SDG treatment. Our results showed that SDG did not further extend the lifespan of *aak-2* mutant strain RB754, but could moderately extend the lifespan of *isp-1* mutant strain MQ887 (7.5%, Figures 6(c) and 6(d)).

## 4. Discussion

Flaxseed is wildly used as both daily food and medicine to strengthen human health. As a phytoestrogen extracted from flaxseed, SDG exhibits many beneficial bioactivities, such as anti-inflammatory, antioxidant, antimutagenic, antiobesity, antihypolipidemic, and neuroprotective effects [8]. So, we are wondering if SDG could delay the aging process of organism. Here, we found that SDG could extend the lifespan of *C. elegans*, increase body movement ability, improve stress resistance, protect DA neurons from 6-OHDA-induced neurodegeneration, and decrease A*β*-induced toxicity in *C. elegans*.

We found that SDG could increase the expression of *hsp-16.2*, *hsp-6*, and *hsp-60* at mRNA level and increase the expression of *hsp-4* and *hsp-6* at the protein level in *C. elegans*. Heat shock proteins (HSPs) are molecular chaperones and have been implicated in longevity and aging in many organisms [47]. Meanwhile, the expression of *hsp-4* and *hsp-6* is a positive marker of unfolded protein response (UPR), which could respond to heat stress [48, 49]. Aging worms show impaired activation of heat shock and UPRs [50]. However, the increased expression of proteins HSP-4 and HSP-6 was not strong enough to show that SDG extend the lifespan of worms via activating UPR. Heat shock factor 1 (HSF-1), an essential nuclear protein, is associated with heat-stress response and aging in *C. elegans* [29]. We also found that SDG could not extend the lifespan of the *hsf-1* mutant, which suggested that SDG may require HSF-1 to extend the lifespan of *C. elegans*.

As a phytoestrogen, SDG is metabolized in the gut to bioactive metabolites enterolactone and enterodiol, both of which can alter estrogen signaling in mammals [51]. Because of estrogen agonist or antagonist properties, flaxseed and its lignan precursor, SDG, affect pregnancy outcome or reproductive development in rats [52]. Moreover, SDG inhibits adipogenesis by activating AMPK*α*, suggesting it could be an attractive therapeutic candidate for the treatment of obesity [53]. We know that fat metabolism and reproduction are associated with aging [54]. Because SDG can regulate the reproductive development and fat metabolism, we are wondering if this activity could extend the lifespan of worms. Nuclear transcriptional factors DAF-16, DAF-12, and NHR-80 have an effect on the regulation of lifespan, stress resistance, lipid metabolism, and reproductive ability in *C. elegans*. We found that the expression levels of DAF-16, DAF-12, and NHR-80 targeted genes were upregulated in *C. elegans* treated with SDG. The expression level of protein SOD-3 was also increased in *C. elegans* treated with SDG. Reduced reproduction is associated with increased fat storage and prolonged lifespan in multiple organisms. However, SDG decreased the fat content of worms without reducing the number of off-spring. The *glp-1* mutant strain shows prolonged lifespan due to failed germline proliferation. SDG shortened the lifespan of the *glp-1* mutant by 10.0%. We hypothesized that SDG may regulate the germline signals in *C. elegans* as a phytoestrogen to compensate the reproductive signaling loss due to the null mutation of *glp-1*. We may need more evidence to verify our hypothesis.

The pumping rate of pharyngeal reduces with aging in *C. elegans* [55]. We found that the pumping rate of the pharyngeal was significantly decreased in *C. elegans* treated with SDG. Reducing pharyngeal pumping often leads to decreased food intake in *C. elegans*, which might result in the activation of the DR signaling pathway. Because reduction of food intake also delays the growth, measuring whether SDG-treated animals grow slower taking longer to reach L4s or adulthoods will serve as an indirect way to address this issue. We further observed that SDG slowed down the growth and development of worms. The unadministered wild-type worm reached the adult stage at 48 h while the administration group reached the same stage at 60 h (Figure S2). In response to nutrient deprivation, animals may curtail energetically expensive processes such as reproduction [56]. However, we did not find that SDG decreased the number of progeny significantly in wild-type. We hypothesized that reduced reproductive capacity due to the activated DR signaling pathway was alleviated by the estrogen effect of SDG. We need more evidence to verify our hypothesis. We further found that SDG could not extend the lifespan of mutants from genes *aak-2* and *eat-2*, while still extending the lifespan of *isp-1* mutant, 6.6%. These results suggested that the effect of SDG on the lifespan extension might not be strong enough to distinguish from the lifespan of these long-lived mutants.

In summary, we found that SDG could increase stress resistance, alleviate dopamine neurodegeneration induced by 6-OHDA, and reduce A*β*-induced toxicity in *C. elegans*. Moreover, SDG extended the lifespan of *C. elegans* via DAF-16 and HSF-1. It is worth to further investigate the beneficial effects of SDG, especially lifespan extension and treatment for age-related disease.

## Data Availability

All the figures and tables used to support the findings of this study are included within the article.

## Supplementary Materials

Supplementary Materials Table S1: mean lifespan of wild-type C. elegans (N2) treated with a series of different concentrations of SDG. Table S2: lifespan extension fold of N2 treated with a series of different concentrations of secoisolariciresinol diglucoside (SDG). Table S3: Effect of SDG on the chemotaxis behavior of N2. Table S4: effect of SDG on body bending. Table S5: effect of SDG on lifespan of C. elegans. Table S6: effect of SDG on mRNA expression in wild-type C. elegans (N2). Table S7: effect of SDG on expressions of HSP-4, HSP-6, and DAT-1. Table S8: effect of SDG on expression of ROS. Table S9: effect of SDG on delaying the time of paralysis. Table S10: effect of SDG on expression of SOD-3. Table S11: effect of SDG on fat content. Table S12: effect of SDG on progeny viability. Table S13: effect of SDG on pharyngeal pumping. Figure S1: the live image of chemotaxis assays. Figure S2: effect of SDG on the growth of C. elegans.
