# Supplementary material for: Secoisolariciresinol Diglucoside Delays the Progression of Aging-Related Diseases and Extends the Lifespan of Caenorhabditis elegans via DAF-16 and HSF-1
Source: Oxid Med Cell Longev. 2020 Jul 14;2020:1293935. doi: 10.1155/2020/1293935 (PMC7378611; doi:10.1155/2020/1293935)
Supplement: Supplementary Materials — Table S1: mean lifespan of wild-type C. elegans (N2) treated with a series of different concentrations of SDG. Table S2: lifespan extension fold of N2 treated with a series of different concentrations of secoisolariciresinol diglucoside (SDG). Table S3: Effect of SDG on the chemotaxis behavior of N2. Table S4: effect of SDG on body bending. Table S5: effect of SDG on lifespan of C. elegans. Table S6: effect of SDG on mRNA expression in wild-type C. elegans (N2). Table S7: effect of SDG on expressions of HSP-4, HSP-6, and DAT-1. Table S8: effect of SDG on expression of ROS. Table S9: effect of SDG on delaying the time of paralysis. Table S10: effect of SDG on expression of SOD-3. Table S11: effect of SDG on fat content. Table S12: effect of SDG on progeny viability. Table S13: effect of SDG on pharyngeal pumping. Figure S1: the live image of chemotaxis assays. Figure S2: effect of SDG on the growth of C. elegans. [file 1293935.f1.docx]

**Secoisolariciresinol diglucoside delays the progression of aging-related diseases and extends the lifespan of *Caenorhabditis elegans* via DAF-16 and HSF-1**

**Min Lu ^1^, Lin Tan ^1^, Xiao-Gang Zhou ^1^, Zhong-Lin Yang ^1^, Qing Zhu ^1^, Jian-Ning Chen ^1^, Huai-Rong Luo ^1, 2, 3,*^, Gui-Sheng Wu ^1, 2, *^**

1 Key Laboratory for Aging and Regenerative Medicine, Department of Pharmacology School of Pharmacy, Southwest Medical University, Luzhou, Sichuan 646000, China.

2 Key Laboratory of Medical Electrophysiology, Ministry of Education, Institute of

Cardiovascular Research of Southwest Medical University, Luzhou, Sichuan 646000, China.

3 Central Nervous System Drug Key Laboratory of Sichuan Province, Luzhou, Sichuan 646000, China

* Corresponding author: Dr. Huai-Rong Luo, Gui-Sheng Wu;

Key Laboratory for Aging and Regenerative Medicine, Department of Pharmacology

School of Pharmacy, Southwest Medical University

319 Zhongshan Road, Luzhou, Sichuan 646000, China

Phone: +86 830-3160842; Fax: +86 830-3160842

E-mail address: [lhr@swmu.edu.cn](mailto:lhr@swmu.edu.cn), [wgs@swmu.edu.cn](mailto:wgs@swmu.edu.cn)

**Supplementary materials**

**Contents:**

**Table S1. Mean lifespan of wild-type *C. elegans* (N2) treated with a serial of different concentrations of SDG**

**Table S2. Lifespan extension fold of N2 treated with a serial of different concentrations of Secoisolariciresinol diglucoside (SDG)**

**Table S3. Effect of SDG on the chemotaxis behavior of N2**

**Table S4. Effect of SDG on body bending**

**Table S5. Effect of SDG on lifespan of *C. elegans***

**Table S6. Effect of SDG on mRNA expresssion in wild-type *C. elegans* (N2)**

**Table S7. Effect of SDG on expressions of HSP-4, HSP-6, and DAT-1**

**Table S8. Effect of SDG on expression of ROS**

**Table S9. Effect of SDG on delaying the time of paralysis**

**Table S10. Effect of SDG on expression of SOD-3**

**Table S11. Effect of SDG on fat content**

**Table S12. Effect of SDG on progeny viability**

**Table S13. Effect of SDG on pharyngeal pumping**

**Figure S1. The live image of chemotaxis assays**

**Figure S2. Effect of SDG on the growth of *C. elegans***

**Table S1. Mean lifespan of wild-type *C. elegans* (N2) treated with a serial of different concentrations of SDG**

| **Figure 1(b)** | **Concentration (μM)** |  | **0** | **50** | **200** | **500** |
| --- | --- | --- | --- | --- | --- | --- |
|  |  |  |  |  |  |  |
|  | **Strain** |  | N2 | N2 | N2 | N2 |
|  | **Treatment** |  | 20 °C/OP50 (dead) | 20 °C/OP50 (dead) | 20 °C/OP50 (dead) | 20 °C/OP50 (dead) |
|  | **Mean lifespan ± SEM**  **(day)** | EXP. 1 | 19.446 ± 0.434 | 20.394 ± 0.501 | 20.922 ± 0.479 | 23.718 ± 0.468 |
|  |  | EXP. 2 | 20.816 ± 0.546 | 24.060 ± 0.488 | 24.612 ± 0.457 | 25.602 ± 0.413 |
|  |  | EXP. 3 | 21.540 ± 0.539 | 24.596 ± 0.480 | 25.293 ± 0.480 | 26.068 ± 0.416 |
|  | ***p* value vs control** | EXP. 1 |  |  | < 0.0001 |  |
|  |  | EXP. 2 |  |  | < 0.001 |  |
|  |  | EXP. 3 |  |  | < 0.0001 |  |
|  | **N** | EXP. 1 | 101 | 88 | 89 | 103 |
|  |  | EXP. 2 | 103 | 100 | 110 | 103 |
|  |  | EXP. 3 | 100 | 94 | 99 | 103 |
|  | **Change in mean lifespan** | EXP. 1 |  | 10.1% | 13.5% | 22.0% |
|  |  | EXP. 2 |  | 15.6% | 18.2% | 23.0% |
|  |  | EXP. 3 |  | 14.2% | 17.4% | 21.0% |

N: the number of dead animals. The mean lifespan values were calculated by a log rank (Mantel-Cox) statistical test. *p* values were calculated for individual experiments, each consisting of control and experimental animals as the same time. All statistical were calculated by using SPSS package.

**Table S2. Lifespan extension fold of N2 treated with a serial of different concentrations of Secoisolariciresinol diglucoside (SDG)**

| **Figure 1(c)** | **Concentration**  **(μM)** | **0** | **50** | **200** | **500** |
| --- | --- | --- | --- | --- | --- |
| **Lifespan extension (%)** | EXP. 1 | 0 | 10.1 | 13.5 | 22.0 |
|  | EXP. 2 | 0 | 15.6 | 18.2 | 23.0 |
|  | EXP. 3 | 0 | 14.2 | 17.4 | 21.0 |

**Table S3. Effect of SDG on the chemotaxis behavior of N2**

| **Figure 1(d)** | **Strain** | **Treatment** | **EXP. 1** | **CI 1** | **EXP. 2** | **CI 2** | **EXP. 3** | **CI 3** | **CI (Mean)** |
| --- | --- | --- | --- | --- | --- | --- | --- | --- | --- |
|  | **N2** | 20 °C/Control | 66 |  | 77 |  | 72 |  |  |
|  |  | 20 °C/50 μM SDG | 72 | 0.043 | 85 | 0.049 | 80 | 0.053 | 0.048 |
|  |  | 20 °C/Control | 67 |  | 64 |  | 90 |  |  |
|  |  | 20 °C/200 μM SDG | 79 | 0.082 | 76 | 0.085 | 107 | 0.086 | 0.085 |
|  |  | 20 °C/Control | 65 |  | 75 |  | 80 |  |  |
|  |  | 20 °C/500 μM SDG | 92 | 0.172 | 105 | 0.167 | 109 | 0.153 | 0.164 |

N: total number of analysis. SDG: secoisolariciresinol diglucoside. CI：chemotaxis index. In each experiment, control and experimental animals were analyzed in parallel.

**Table S4. Effect of SDG on body bending**

| **Figure 1(e)** | **Strain** | **Treatment** | **Number of body bending (per 20 seconds)**  **Mean ± SEM** | ***p* value vs control** | **N** | **Number of body bending (per 20 seconds)**  **Mean ± SEM** | ***p* value vs control** | **N** |
| --- | --- | --- | --- | --- | --- | --- | --- | --- |
|  | **N2** | **OP50 (dead)** | **5 d** | | | **10 d** | | |
|  | EXP. 1 | 20 °C/Control | 24 ± 0.682 | <0.0001 | 26 | 16 ± 0.478 | < 0.0001 | 27 |
|  | EXP. 1 | 20 °C/500 μM SDG | 30 ± 0.756 |  | 26 | 25 ± 0.561 |  | 27 |
|  | EXP. 2 | 20 °C/Control | 30 ± 0.473 | <0.0001 | 30 | 25 ± 0.681 | < 0.0001 | 30 |
|  | EXP. 2 | 20 °C/500 μM SDG | 41 ± 0.726 |  | 30 | 37 ± 0.745 |  | 30 |
|  | EXP. 3 | 20 °C/Control | 28 ± 0.611 | < 0.001 | 31 | 23 ± 0.495 | < 0.0001 | 32 |
|  | EXP. 3 | 20 °C/500 μM SDG | 33 ± 0.373 |  | 31 | 31 ± 0.703 |  | 32 |

N: total number of analysis. SDG: secoisolariciresinol diglucoside. Body bending assay were carried out at 20 °C. Body bending was assessed by observation for 20 sec. petri dishes were tapped against the microscope stage to stimulate movement before scoring. *t*-test was used for statistical analysis. In each experiment, control and experimental animals were analyzed in parallel.

**Table S5. Effect of SDG on lifespan of *C. elegans***

|  | **Strain** | **Treatment** | **Mean lifespan ± SEM** | ***p* value vs control** | **Change in mean lifespan** | **N** |
| --- | --- | --- | --- | --- | --- | --- |
|  | **N2** | **OP50 (dead)** | **Day** |  |  |  |
|  | EXP. 1 | 20 °C/Control | 20.116 ± 0.576 | < 0.0001 | 21.8% | 69 |
|  | EXP. 1 | 20 °C/500 μM SDG | 24.507 ± 0.510 |  |  | 75 |
|  | EXP. 2 | 20 °C/Control | 21.114 ± 0.589 | < 0.0001 | 20.3% | 79 |
|  | EXP. 2 | 20 °C/500 μM SDG | 25.390 ± 0.593 |  |  | 59 |
|  | EXP. 3 | 20 °C/Control | 20.782 ± 0.556 | < 0.0001 | 19.7% | 78 |
|  | EXP. 3 | 20 °C/500 μM SDG | 24.868 ± 0.525 |  |  | 68 |
| **Figure 2(a)** | **N2** | **OP50 (dead)** | **Hour** |  |  |  |
|  | EXP. 1 | 35 °C/control | 8.838 ± 0.306 | < 0.0001 | 31.6% | 103 |
|  | EXP. 1 | 35 °C/500μM SDG | 11.632 ± 0.294 |  |  | 101 |
|  | EXP. 2 | 35 °C/control | 7.111 ± 0.259 | < 0.0001 | 33.4% | 90 |
|  | EXP. 2 | 35 °C/500μM SDG | 9.486 ± 0.296 |  |  | 74 |
|  | EXP. 3 | 35 °C/control | 6.986 ± 0.231 | < 0.0001 | 27.7% | 73 |
|  | EXP. 3 | 35 °C/500μM SDG | 8.918 ± 0.169 |  |  | 85 |
| **Figure 3(b)** | **N2** | **20 mM paraquat**  **OP50 (dead)** | **Day** |  |  |  |
|  | EXP. 1 | 20 °C/Control | 4.550 ± 0.230 | < 0.0001 | 27.0% | 60 |
|  | EXP. 1 | 20 °C/500 μM SDG | 5.776 ± 0.277 |  |  | 67 |
|  | EXP. 2 | 20 °C/Control | 4.443 ± 0.220 | 0.001 | 22.5% | 61 |
|  | EXP. 2 | 20 °C/500 μM SDG | 5.443 ± 0.242 |  |  | 70 |
|  | EXP. 3 | 20 °C/Control | 4.484 ± 0.212 | 0.001 | 26.0% | 66 |
|  | EXP. 3 | 20 °C/500 μM SDG | 5.648 ± 0.228 |  |  | 71 |
| **Figure 3(c)** | ***hsf-1* (PS3551)** | **OP50 (dead)** | **Day** |  |  |  |
|  | EXP. 1 | 20 °C/Control | 19.280 ± 0.395 | 0.514 | # | 75 |
|  | EXP. 1 | 20 °C/500 μM SDG | 19.541 ± 0.393 |  |  | 74 |
|  | EXP. 2 | 20 °C/Control | 18.000 ± 0.421 | 0.114 | # | 99 |
|  | EXP. 2 | 20 °C/500 μM SDG | 18.988 ± 0.422 |  |  | 81 |
|  | EXP. 3 | 20 °C/Control | 18.516 ± 0.580 | 0.609 | # | 62 |
|  | EXP. 3 | 20 °C/500 μM SDG | 20.151 ± 0.383 |  |  | 53 |
| **Figure 4(d)** | **CL4176** | **OP50 (dead)** | **Day** |  |  |  |
|  | EXP. 1 | 25 °C/Control | 4.879 ± 0.331 | < 0.0001 | 57.0% | 66 |
|  | EXP. 1 | 25 °C/500 μM SDG | 7.662 ± 0.398 |  |  | 71 |
|  | EXP. 2 | 25 °C/Control | 5.106 ± 0.350 | < 0.0001 | 55.2% | 66 |
|  | EXP. 2 | 25 °C/500 μM SDG | 7.922 ± 0.384 |  |  | 77 |
|  | **Strain** | **Treatment** | **Mean lifespan ± SEM** | ***p* value vs control** | **Change in mean lifespan** | **N** |
|  | EXP. 3 | 25 °C/Control | 4.831 ± 0.335 | <0.0001 | 62.8% | 65 |
|  | EXP. 3 | 25 °C/500 μM SDG | 7.864 ± 0.425 |  |  | 66 |
| **Figure 5(e)** | ***daf-16***  **(CF1038)** | **OP50 (dead)** | **Day** |  |  |  |
|  | EXP. 1 | 20 °C/Control | 19.000 ± 0.525 | 0.648 | # | 63 |
|  | EXP. 1 | 20 °C/500 μM SDG | 18.379 ± 0.368 |  |  | 66 |
|  | EXP. 2 | 20 °C/Control | 18.889 ± 0.538 | 0.806 | # | 63 |
|  | EXP. 2 | 20 °C/500 μM SDG | 19.033 ± 0.560 |  |  | 61 |
|  | EXP. 3 | 20 °C/Control | 19.431 ± 0.532 | 0.537 | # | 65 |
|  | EXP. 3 | 20 °C/500 μM SDG | 18.471 ± 0.497 |  |  | 70 |
|  |  |  |  |  |  |  |
| **Figure 5(f)** | ***daf-12* (AA89)** | **OP50 (dead)** | **Day** |  |  |  |
|  | EXP.1 | 20 °C/Control | 13.652 ± 0.232 | 0.121 | # | 66 |
|  | EXP.1 | 20 °C/500 μM SDG | 13.148 ± 0.226 |  |  | 61 |
|  | EXP.2 | 20 °C/Control | 13.284 ± 0.209 | 0.997 | # | 74 |
|  | EXP.2 | 20 °C/500 μM SDG | 13.290 ± 0.225 |  |  | 62 |
|  | EXP.3 | 20 °C/Control | 13.060 ± 0.220 | 0.937 | # | 67 |
|  | EXP.3 | 20 °C/500 μM SDG | 12.898 ± 0.219 |  |  | 72 |
| **Figure 5(g)** | ***nhr-80***  **(BX165)** | **OP50 (dead)** | **Day** |  |  |  |
|  | EXP.1 | 20 °C/Control | 19.276 ± 0.366 | 0.816 | # | 116 |
|  | EXP.1 | 20 °C/500 μM SDG | 18.796 ± 0.369 |  |  | 142 |
|  | EXP.2 | 20 °C/Control | 18.128 ± 0.513 | 0.087 | # | 94 |
|  | EXP.2 | 20 °C/500 μM SDG | 19.568 ± 0.513 |  |  | 111 |
|  | EXP.3 | 20 °C/Control | 18.555 ± 0.460 | 0.329 |  | 116 |
|  | EXP.3 | 20 °C/500 μM SDG | 18.433 ± 0.451 |  | # | 113 |
| **Figure 5(h)** | ***glp-1***  **(CF1903)** | **OP50 (dead)** | **Day** |  |  |  |
|  | EXP.1 | 20 °C/Control | 27.714 ± 0.575 | < 0.0001 | -11.54% | 84 |
|  | EXP.1 | 20 °C/500 μM SDG | 24.515 ± 0.492 |  |  | 86 |
|  | EXP.2 | 20°C/Control | 27.708 ± 0.518 | < 0.0001 | -9.98% | 89 |
|  | EXP.2 | 20 °C/500 μM SDG | 24.942 ± 0.458 |  |  | 69 |
|  | EXP.3 | 20 °C/500 μM SDG | 26.986 ± 0.637 | < 0.0001 | -8.43% | 72 |
|  | EXP.3 | 20 °C/500 μM SDG | 24.710 ± 0.358 |  |  | 107 |
|  | **Strain** | **Treatment** | **Mean lifespan ± SEM** | ***p* value vs control** | **Change in mean lifespan** | **N** |
| **Figure 6(b)** | ***eat-2***  **(DA1116)** | **OP50 (dead)** | **Day** |  |  |  |
|  | EXP. 1 | 20 °C/Control | 24.013 ± 0.688 | 0.295 |  | 77 |
|  | EXP. 1 | 20 °C/500 μM SDG | 24.844 ± 0.778 |  | # | 64 |
|  | EXP. 2 | 20 °C/Control | 23.875 ± 0.611 | 0.654 |  | 72 |
|  | EXP. 2 | 20 °C/500 μM SDG | 23.787 ± 0.543 |  | # | 75 |
|  | EXP. 3 | 20 °C/Control | 22.803 ± 0.606 | 0.975 |  | 76 |
|  | EXP. 3 | 20 °C/500 μM SDG | 22.400 ± 0.650 |  | # | 75 |
| **Figure 6(c)** | ***aak-2***  ***(*RB754)** | **OP50 (dead)** | **Day** |  |  |  |
|  | EXP. 1 | 20 °C/Control | 21.378 ± 0.428 | 0.915 |  | 74 |
|  | EXP. 1 | 20 °C/500 μM SDG | 21.792 ± 0.380 |  | # | 72 |
|  | EXP. 2 | 20°C/Control | 21.038 ± 0.310 | 0.937 |  | 105 |
|  | EXP. 2 | 20 °C/500 μM SDG | 21.031 ± 0.343 |  | # | 96 |
|  | EXP. 3 | 20°C/Control | 20.173 ± 0.463 | 0.1 |  | 127 |
|  | EXP. 3 | 20 °C/500 μM SDG | 19.443 ± 0.458 |  | # | 106 |
| **Figure 6(d)** | ***isp-1*(MQ887)** | **OP50 (dead)** | **Day** |  |  |  |
|  | EXP. 1 | 20 °C/Control | 21.985 ± 0.390 | 0.018 |  | 66 |
|  | EXP. 1 | 20 °C/500 μM SDG | 23.083 ± 0.427 |  | 5.0% | 72 |
|  | EXP. 2 | 20°C/Control | 21.453 ± 0.308 | 0.013 |  | 86 |
|  | EXP. 2 | 20 °C/500 μM SDG | 23.053 ± 0.420 |  | 7.5% | 76 |
|  | EXP. 3 | 20°C/Control | 21.815 ± 0.388 | 0.001 |  | 65 |
|  | EXP. 3 | 20 °C/500 μM SDG | 23.250 ± 0.460 |  | 6.6% | 68 |

N: the number of dead animals. SDG: secoisolariciresinol diglucoside. #: no calculate (because *P*>0.05).The mean lifespan values were calculated by a log rank (Mantel-Cox) statistical test. *p* values were calculated for individual experiments, each consisting of control and experimental animals as the same time. All statistical were calculated by using SPSS package.

**Table S6. Effect of SDG on mRNA expresssion in wild-type *C. elegans* (N2)**

|  | **Gene** | **EXP. 1** | **EXP. 2** | **Mean** | ***p* value vs control** |
| --- | --- | --- | --- | --- | --- |
|  | ***Control*** | 1 | 1 | 1 |  |
| **Figure 2(b)** | ***hsp-60*** | 1.605 | 1.498 | 1.552 | 0.0093 |
|  | ***hsp-6*** | 1.326 | 1.324 | 1.325 | < 0.0001 |
|  | ***hsp-16.2*** | 2.272 | 2.052 | 2.162 | 0.0088 |
| **Figure 5(a)** | ***sod-3*** | 1.940 | 1.999 | 1.970 | 0.0009 |
|  | ***fard-1*** | 2.268 | 2.446 | 2.357 | 0.0043 |
|  | ***fat-6*** | 1.537 | 1.498 | 1.518 | 0.0014 |

**Table S7. Effect of SDG on expressions of HSP-4, HSP-6, and DAT-1**

|  | **Strain** | **Treatment** | **Fluorescence intensity**  **Mean ± SEM** | ***p* value vs control** | **N** |
| --- | --- | --- | --- | --- | --- |
| **Figure 2(c)** | **SJ4005**  ***(hsp-4::gfp)*** | **OP50 (dead)** |  |  |  |
|  | EXP. 1 | 20 °C/Control | 4.343 ± 0.368 | < 0.0001 | 38 |
|  | EXP. 1 | 20 °C/500 μM SDG | 8.946 ± 0.562 |  | 38 |
|  | EXP. 2 | 20 °C/Control | 6.391 ± 0.211 | < 0.0001 | 30 |
|  | EXP. 2 | 20 °C/500 μM SDG | 10.156 ± 0.448 |  | 30 |
|  | EXP. 3 | 20 °C/Control | 6.708 ± 0.213 | < 0.0001 | 34 |
|  | EXP. 3 | 20 °C/500 μM SDG | 11.093 ± 0.681 |  | 34 |
| **Figure 2(c)** | **SJ4100 *(hsp-6::gfp)*** | **OP50 (dead)** |  |  |  |
|  | EXP. 1 | 20 °C/Control | 9.701 ± 0.293 | < 0.0001 | 38 |
|  | EXP. 1 | 20 °C/200 μM SDG | 14.953 ± 0.593 |  | 38 |
|  | EXP. 2 | 20 °C/Control | 9.738 ± 0.552 | < 0.0001 | 41 |
|  | EXP. 2 | 20 °C/500 μM SDG | 15.120 ± 0.537 |  | 41 |
|  | EXP. 3 | 20 °C/Control | 9.701 ± 0.293 | < 0.0001 | 35 |
|  | EXP. 3 | 20 °C/500 μM SDG | 14.953 ± 0.593 |  | 35 |
| **Figure 4(a)** | **BZ555**  ***(dat-1::gfp)*** | **OP50 (dead)** |  |  |  |
|  | EXP. 1 | 20 °C/Control | 26.859 ± 0.798 | < 0.0001 | 33 |
|  | EXP. 1 | 20 °C/6-OHDA | 14.476 ± 0.690 |  | 33 |
|  | EXP. 1 | 20 °C/6-OHDA  +500 μM SDG | 23.591 ± 1.221 |  | 33 |
|  | EXP. 2 | 20 °C/Control | 26.206 ± 0.774 | < 0.0001 | 33 |
|  | EXP. 2 | 20 °C/6-OHDA | 14.435 ± 0.563 |  | 33 |
|  | EXP. 2 | 20 °C/6-OHDA  +500 μM SDG | 22.927 ± 1.171 |  | 33 |
|  | EXP. 3 | 20 °C/Control | 24.626 ± 0.478 | < 0.0001 | 36 |
|  | EXP. 3 | 20 °C/6-OHDA | 13.998 ± 0.591 |  | 36 |
|  | EXP. 3 | 20 °C/6-OHDA  +500 μM SDG | 22.176 ± 1.193 |  | 36 |

N: total number of analysis. SDG: secoisolariciresinol diglucoside. *t*-test was used for statistical analysis. In each experiment, control and experimental animals were analyzed in parallel.

**Table S8. Effect of SDG on expression of ROS**

| **Figure 3(a)** | **Date** | **Strain** |  |  |
| --- | --- | --- | --- | --- |
|  |  | **N2**  **(wild-type)** |  |  |
| **Treatment** |  |  | **OP50 (dead)**  **20 °C/Control** | **OP50 (dead)**  **20 °C/500 μM SDG** |
| **Fluorescence intensity**  **Mean ± SEM** | **5 d** | EXP. 1  EXP. 2  EXP. 3 | 7.380 ± 0.147  7.335 ± 0.116  7.438 ± 0.152 | 5.281 ± 0.084  5.205 ± 0.090  5.388 ± 0.074 |
| ***p* value vs control** |  | EXP. 1  EXP. 2  EXP. 3 |  | <0.0001  <0.0001  <0.0001 |
| **N** |  | EXP. 1  EXP. 2  EXP. 3 | 30  30  30 | 30  30  30 |
| **Fluorescence intensity**  **Mean ± SEM** | **10 d** | EXP. 1  EXP. 2  EXP. 3 | 16.106 ± 0.557  16.177 ± 0.527  16.237 ± 0.544 | 11.998 ± 0.375  12.040 ± 0.381  12.097 ± 0.377 |
| ***p* value vs control** |  | EXP. 1  EXP. 2  EXP. 3 |  | < 0.0001  < 0.0001  < 0.0001 |
| **N** |  | EXP. 1  EXP. 2  EXP. 3 | 30  30  30 | 30  30  30 |

N: total number of analysis. SDG: secoisolariciresinol diglucoside. *t*-test was used for statistical analysis. In each experiment, control and experimental animals were analyzed in parallel.

**Table S9. Effect of SDG on delaying the time of paralysis**

| **Figures. 4(b) and 4(c)** | **Strain** | **Treatment** | **Time of paralysis**  **Mean ± SEM (hour)** | ***p* value vs control** | **Change in time of paralysis** | **N** |
| --- | --- | --- | --- | --- | --- | --- |
|  | **CL4176** | **OP50 (dead)** |  |  |  |  |
|  | EXP. 1 | 25 °C/Control | 43.952 ± 0.526 | < 0.0001 | 9.5% | 59 |
|  | EXP. 1 | 25 °C/500 μM SDG | 48.130 ± 0.572 |  |  | 84 |
|  | EXP. 2 | 25 °C/Control | 44.034 ± 0.609 | < 0.0001 | 9.0% | 83 |
|  | EXP. 2 | 25 °C/500 μM SDG | 48.000 ± 0.547 |  |  | 92 |
|  | EXP. 3 | 25 °C/Control | 43.385 ± 0.545 | < 0.0001 | 9.5% | 138 |
|  | EXP. 3 | 25 °C/500 μM SDG | 47.487 ± 0.640 |  |  | 118 |

N: the number of paralyzed animals. SDG: secoisolariciresinol diglucoside. The mean values were calculated by a log rank (Mantel-Cox) statistical test. *p* value were calculated for individual experiments, each consisting of control and experimental animals as the same time. All statistical were calculated by using SPSS package.

**Table S10. Effect of SDG on expression of SOD-3**

| **Figure 5(b)** | **Date** | **Strain** |  |  |
| --- | --- | --- | --- | --- |
|  |  | **CF1553 *(sod-3::gfp)*** |  |  |
| **Treatment** |  |  | **OP50 (dead)**  **20 °C/Control** | **OP50 (dead)**  **20 °C/500 μM SDG** |
| **Fluorescence intensity**  **Mean ± SEM** | **5 d** | EXP. 1  EXP. 2  EXP. 3 | 16.853 ± 0.726  16.918 ± 0.583  16.406 ± 0.636 | 20.536 ± 0.782  20.703 ± 0.847  20.188 ± 0.655 |
| ***p* value vs control** |  | EXP. 1  EXP. 2  EXP. 3 |  | 0.001  0.0005  0.0006 |
| **N** |  | EXP. 1  EXP. 2  EXP. 3 | 35  31  30 | 30  30  30 |
| **Fluorescence intensity**  **Mean ± SEM** | **10 d** | EXP. 1  EXP. 2  EXP. 3 | 6.641 ± 0.442  6.873 ± 0.583  6.206 ± 0.560 | 10.072 ± 0.679  10.221 ± 0.620  10.143 ± 0.466 |
| ***p* value vs control** |  | EXP. 1  EXP. 2  EXP. 3 |  | < 0.0001  < 0.0001  < 0.0001 |
| **N** |  | EXP. 1  EXP. 2  EXP. 3 | 30  30  30 | 30  30  30 |

N: total number of analysis. SDG: secoisolariciresinol diglucoside. *t*-test was used for statistical analysis. In each experiment, control and experimental animals were analyzed in parallel.

**Table S11. Effect of SDG on fat content**

| **Figure 5(c)** | **Strain** | **Treatment** | **gray value**  **Mean ± SEM** | ***p* value vs control** | **N** |
| --- | --- | --- | --- | --- | --- |
|  | N2 | OP50(dead) |  |  |  |
|  | EXP. 1 | 20 °C/Control | 116.149 ± 0.884 | <0.0001 | 48 |
|  | EXP. 1 | 20 °C/500 μM SDG | 102.136 ± 1.337 |  | 48 |
|  | EXP. 2 | 20 °C/Control | 116.425 ± 1.162 | <0.0001 | 39 |
|  | EXP. 2 | 20 °C/500 μM SDG | 103.533 ± 1.402 |  | 39 |
|  | EXP. 3 | 20 °C/Control | 117.018 ± 0.972 | <0.0001 | 36 |
|  | EXP. 3 | 20 °C/500 μM SDG | 103.336 ± 1.591 |  | 36 |

N: total number of analysis. SDG: secoisolariciresinol diglucoside. *t*-test was used for statistical analysis. In each experiment, control and experimental animals were analyzed in parallel.

**Table S12. Effect of SDG on progeny viability**

| **Figure 5(d)** | **time** |  |  |  | ***p* value vs control** |
| --- | --- | --- | --- | --- | --- |
| **Strain** |  |  | **N2** | **N2** |  |
| **Treatment** |  |  | **20 °C/Control**  **OP50(dead)** | **20 °C/500 μM SDG**  **OP50(dead)** |  |
| **Number of progeny**  **Mean ± SEM** | 1d | EXP. 1  EXP. 2  EXP. 3 | 29 ± 3.778  22 ± 1.242  17 ± 1.153 | 28 ±3.439  29 ± 1.230  18 ± 0.913 |  |
|  | 2d | EXP. 1  EXP. 2  EXP. 3 | 55 ± 2.908  61 ± 3.481  52 ± 2.587 | 50 ± 2.050  52 ± 2.755  52 ± 2.093 |  |
|  | 3d | EXP. 1  EXP. 2  EXP. 3 | 50 ± 2.884  45 ± 1.678  67 ± 2.529 | 51 ± 3.890  47 ± 1.763  59 ± 2.235 |  |
|  | 4d | EXP. 1  EXP. 2  EXP. 3 | 30 ± 1.738  35 ± 2.690  43 ± 2.650 | 27 ± 1.777  34 ± 2.117  37 ± 2.153 |  |
|  | 5d | EXP. 1  EXP. 2  EXP. 3 | 14 ± 1.214  15 ± 1.840  7 ± 0.949 | 13± 1.570  15± 1.190  11 ±1.040 |  |
|  | 6d | EXP. 1  EXP. 2  EXP. 3 | 3 ± 0.489  2 ± 0.413  2 ± 0.473 | 3 ± 0.545  2 ± 0.385  2 ± 0.434 |  |
| **Total number of progeny** |  | EXP. 1  EXP. 2  EXP. 3 | 180 ± 5.379  185 ± 5.377  187 ± 4.570 | 171 ± 4.570  178 ± 4.640  179 ± 5.379 | > 0.05  > 0.05  > 0.05 |

SDG: secoisolariciresinol diglucoside. *t*-test was used for statistical analysis. In each experiment, control and experimental animals were analyzed in parallel.

**Table S13. Effect of SDG on pharyngeal pumping**

| **Figure 6(a)** | **Strain** | **Treatment** | **Number of pharyngeal pumping (per 20 seconds)**  **Mean ± SEM** | ***p* value vs control** | **N** | **Number of pharyngeal pumping (per 20 seconds)**  **Mean ± SEM** | ***p* value vs control** | **N** |
| --- | --- | --- | --- | --- | --- | --- | --- | --- |
|  | **N2** | **OP50 (dead)** | **5 d** | | | **10 d** | | |
|  | EXP. 1 | 20 °C/Control | 45 ±0.644 | < 0.0001 | 31 | 43 ± 0.433 | < 0.0001 | 32 |
|  | EXP. 1 | 20 °C/500 μM SDG | 36 ± 0.670 |  | 31 | 32 ± 0.487 |  | 32 |
|  | EXP. 2 | 20 °C/Control | 44 ± 0.444 | < 0.0001 | 30 | 43 ± 0.462 | < 0.0001 | 30 |
|  | EXP. 2 | 20 °C/500 μM SDG | 34 ± 0.858 |  | 30 | 31 ± 0.733 |  | 30 |
|  | EXP. 3 | 20 °C/Control | 45 ± 0.982 | < 0.0001 | 30 | 44 ± 0.835 | <0.0001 | 30 |
|  | EXP. 3 | 20 °C/500 μM SDG | 38 ± 0.878 |  | 30 | 34 ± 0.930 |  | 30 |

N: total number of analysis. SDG: secoisolariciresinol diglucoside. *t*-test was used for statistical analysis. In each experiment, control and experimental animals were analyzed in parallel.

**Figure S1. The live image of chemotaxis assays**

**(a)**

**
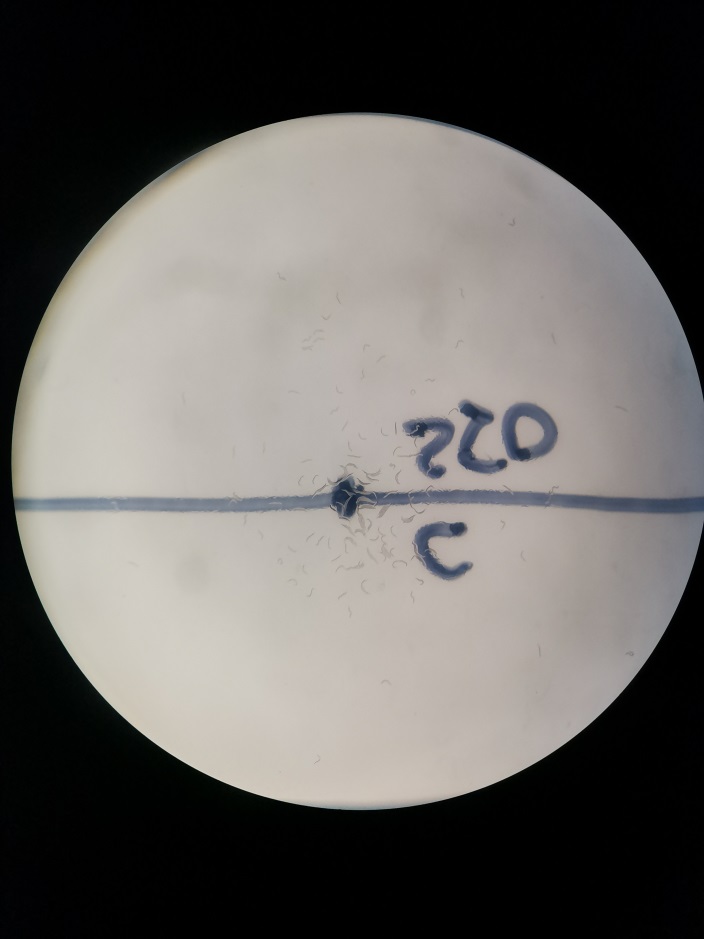
**

50 μM of SDG + 2% tetramisole hydrochloride

H_2_O + 2% tetramisole hydrochloride

**(b)**

**
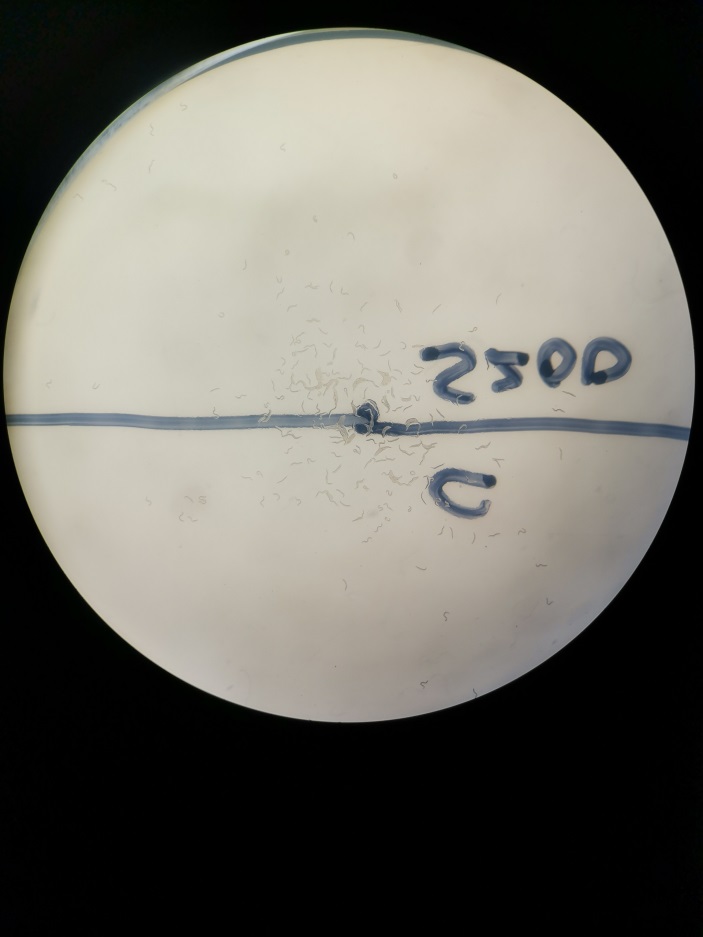
**

200 μM of SDG + 2% tetramisole hydrochloride

H_2_O + 2% tetramisole hydrochloride

**(c)**

**
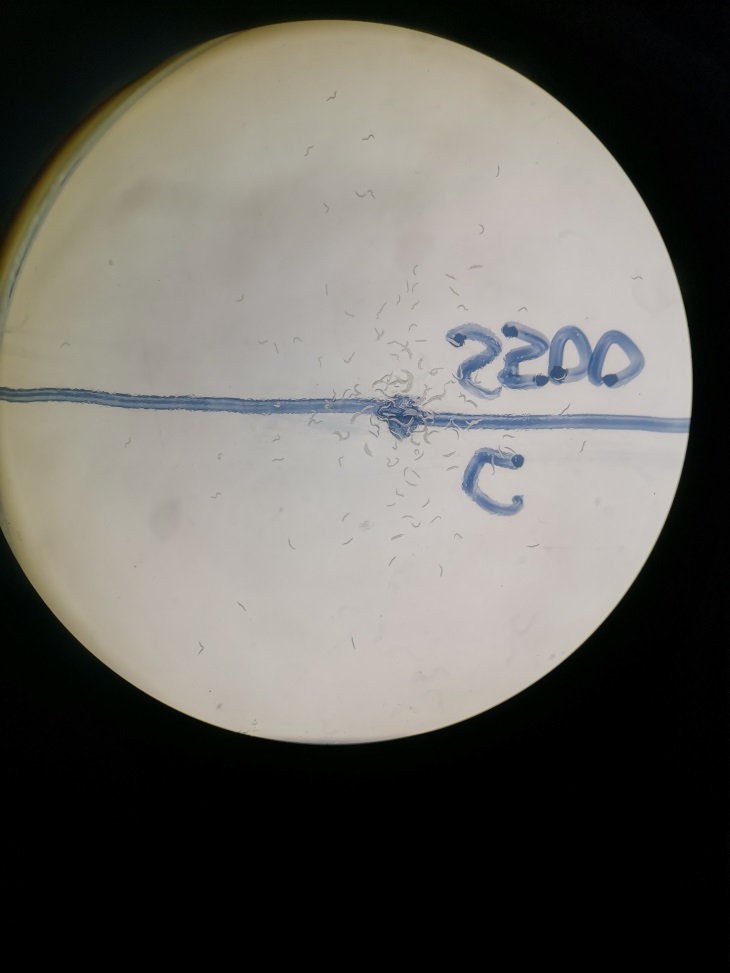
**

500 μM of SDG + 2% tetramisole hydrochloride

H_2_O + 2% tetramisole hydrochloride

Figure S1. The live image of chemotaxis assays. (**a)** The picture of worms treated with or without 50 μM of SDG. **(b)** The picture of worms treated with or without 200 μM of SDG. (**c)** The picture of worms treated with or without 500 μM of SDG. We observed that *C. elegans* did not use chemotaxis to avoid SDG.

**Figure S2. Effect of SDG on the growth of *C. elegans***

**(a)**

**
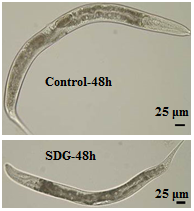
**

**V**

**D**

**A**

**P**

**D**

**V**

**P**

**A**

**(b)**

**
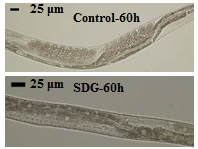
**

**D**

**V**

**A**

**P**

**V**

**D**

**A**

**P**

**(c)**

**
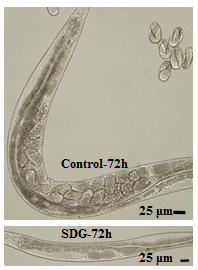
**

**V**

**D**

**A**

**P**

**D**

**V**

**A**

**P**

Figure S2. Effect of SDG on the growth of *C. elegans*. (**a)** The picture of worms treated with or without 500 μM of SDG for 48h. **(b)** The picture of worms treated with or without 500 μM of SDG for 60h. (**c)** The picture of worms treated with or without 500 μM of SDG for 72h. We observed that SDG could delay the growth of *C. elegans*.
